# Transcriptional Cytokine Responses Linked to Pathological Outcomes in Broiler Chickens Fed Residual Doses of Antimicrobials

**DOI:** 10.3390/ijms27135690

**Published:** 2026-06-24

**Authors:** Anna Slawinska, Joanna Bogucka, Aleksandra Dunislawska, Sebastian Knaga, Maria Siwek, Agnieszka Chłodowska, Monika Olszewska-Tomczyk, Malgorzata Olejnik

**Affiliations:** 1 Department of Basic and Preclinical Sciences, Institute of Veterinary Medicine, Faculty of Biological and Veterinary Sciences, Nicolaus Copernicus University, Szosa Bydgoska 13, 87-100 Toruń, Poland; 2 Department of Animal Biotechnology and Genetics, Faculty of Animal Breeding and Biology, Bydgoszcz University of Science and Technology, Mazowiecka 28, 85-084 Bydgoszcz, Poland; 3 Department of Infectious and Invasive Diseases and Veterinary Administration, Institute of Veterinary Medicine, Faculty of Biological and Veterinary Sciences, Nicolaus Copernicus University, Szosa Bydgoska 13, 87-100 Toruń, Poland

**Keywords:** antibiotic residues, feed cross-contamination, cytokine, mucin, immune modulation

## Abstract

Cross-contamination of poultry feed with antimicrobials may cause unintended exposure to residual antibiotic doses, but the immunological consequences remain unclear. We tested whether six antimicrobials (colistin, doxycycline, flumequine, thiamphenicol, tiamulin, and tilmicosin) delivered in feed at 2% of the maximum approved dose modulate immune-related gene expression and splenic histology in broiler chickens. Female Ross 308 chicks received antibiotic-contaminated diets throughout the rearing. Spleen and caecal mucosa were sampled on days 7, 21, and 35, and analyzed by RT-qPCR. Spleen was sampled on day 35 for histopathology. Responses were strongly time- and tissue-dependent, with the largest effects on day 21. In spleen, selected antimicrobials showed selective down-regulation of *IL-2*, *IL-4*, *IL-6*, *IL-8*, *IL-12p40*, and *IFN-ß*, while *IFN-γ* was significantly up-regulated (*p* ≤ 0.05). In caecal mucosa, transcriptional modulation was weaker and more limited. Significant changes were restricted to *MUC2* on day 21 and to *IL-2* and *IL-8* on day 35 (*p* ≤ 0.05). Splenic histopathology showed unchanged lymphatic nodule counts, but increased lymphoid atrophy, necrosis, and inflammatory infiltrates, most frequently in colistin- and doxycycline-fed birds. Overall, residual antimicrobials in feed are not biologically neutral and are associated with distinct structural changes in immune-related gene expression and splenic tissue development.

## 1. Introduction

Although antimicrobials are administered to food-producing animals mainly via oral solutions, the medicated premixes remain an important means of treatment, reaching 17.1% of total sales of antimicrobial Veterinary Medicinal Products (VMPs) in 2023 [1]. As many as 24 antimicrobials are approved to be incorporated in the premixes in the European Union. Their importance for human medicine differs greatly, but some of them have been even assigned to B category “restrict” according to Antimicrobial Advice ad hoc Expert Group (AMEG). These include polymyxins (colistin) and fluoroquinolones (flumequine and oxolinic acid) [2]. The administration of those antimicrobials is strongly discouraged by scientific guidelines. However, they are still authorized for use in many European countries. Therefore, the risks associated with their non-prudent use cannot be discarded.

The cross-contamination of the feed with veterinary antibiotics poses a risk factor of uncontrolled exposure of the animals and their microbiome. The residuals of antibiotics are delivered to animals as contaminants of the farm environment via feed or water. The cross-contamination can occur during manufacturing and processing of the unmedicated feed using the same equipment as for medicated feed. Therefore, the sources of cross-contamination of the feed are attributed to the feed mills, transport trucks, and storage on the farm [3]. The legislation of the European Union addressed this issue by implementing the maximum level for non-medicated feed as 1% of the amount of active substance that is allowed in medicated feed [4].

Orally administered antimicrobials pass through the gastrointestinal tract and come into contact with the resident microbiota. Depending on the specific antimicrobial, they may be absorbed, transformed by the enzymes of the host and/or microorganisms, excreted, or released back into the gut [5]. As a result, antimicrobial exposure and local drug concentrations in the gastrointestinal tract may directly affect gut microbial communities. Even low doses of antimicrobials can create selective pressure favoring resistant bacteria [6]. Antimicrobials may also modulate physiological and immune functions of the host, either directly or indirectly through changes in the gut microbiota. In chickens, the most available evidence on the effects of low antimicrobial doses on the immune responses comes from studies on antibiotic growth promoters (AGPs), which were historically administered at subtherapeutic levels. For example, AGPs have been reported to modulate cytokine and chemokine expression [7,8], antibody responses [9], and immune-cell populations [10] in chickens. However, it remains insufficiently understood whether residual antimicrobial doses resulting from feed cross-contamination can similarly modulate immune responses.

Antibiotics have direct and indirect immunomodulatory effects. Direct effects involve interactions between the antimicrobial compound and host immune cells, resulting in modulation of immune-cell signaling or effector functions. Indirect effects are primarily related to antimicrobial-induced changes in microbiota composition and function, which may influence host immune responses [11]. Much of the evidence for direct immunomodulatory effects comes from in vitro or ex vivo studies, and the effects vary among antimicrobial classes and experimental model. In turn, in vivo studies allow assessment of the integrated immune response resulting from direct drug effects, microbiota-mediated mechanisms, and host physiological context.

Colistin has been reported to exert complex immunomodulatory effects. In cell-based models, low concentrations reduced the expression of pro-inflammatory cytokines (*IL-1β*, *TNF-α*, *IL-6*, and *IL-8*) and increased IL-10 in stimulated human mononuclear cells [12]. In another experimental model it enhanced macrophage activity and increased TNF-α, IL-1β, and IL-6 secretion [13]. Doxycycline appears to act more consistently as an anti-inflammatory agent, reducing LPS-induced *TNF-α*, *IL-6*, and *IL-8* expression, as well as inflammasome-related responses [14,15]. The immunomodulatory activity of fluoroquinolones is also well recognized [16,17]. Although flumequine has been studied less extensively, it has been shown to affect the expression of CD3+, CD4+, and CD8+ T-cell markers in lymphoid tissues [18]. Data on thiamphenicol are limited, but available studies suggest that it may influence innate immune-cell functions [19,20]. Tiamulin has also been reported to exert anti-inflammatory effects through inhibition of TNF-α and suppression of NF-κB and MAPK signaling [21] and may additionally affect post-vaccinal immune responses [22]. Tilmicosin reduced the production of TNF-α, IL-1β, and IL-6, while increasing IL-10 in LPS-stimulated macrophages and monocytes [23]. In chickens, tilmicosin administration during Newcastle disease vaccination reduced early antibody production, indicating that it may also affect adaptive immune responses in birds [24]. Together, these findings show that several antimicrobial classes can modulate immune signaling beyond their direct antibacterial activity.

The aim of this study was to determine whether in-feed exposure to residual doses of six antimicrobials (colistin, doxycycline, flumequine, thiamphenicol, tiamulin, and tilmicosin) modulates immune responses in broiler chickens. To address this, we evaluated cytokine-related gene expression in the spleen and caecal mucosa, together with histopathological changes in splenic tissue.

## 2. Results

### 2.1. Immune-Related Gene Expression

#### 2.1.1. Spleen

Figure 1 presents the relative gene expression of the immune-related genes determined in the spleen of chickens exposed to residual antibiotic doses on days 7, 21, and 35 of rearing. On day 7, transcriptional responses in the spleen were limited, with only sporadic significant differences compared with the control. Early responses were characterized by the slight up-regulation of the pro-inflammatory cytokine *IL-1ß* in group 6 (*p* Holm ≤ 0.05), and *IL-4* in group 3 (*p* Holm ≤ 0.05). The only gene that expressed a low but significant down-regulation in comparison to the control was *IFN-γ* in group 1 (*p* Holm ≤ 0.05).

On day 21, the most pronounced transcriptional changes were observed in the spleen, but the significant effects were gene- and treatment-specific. Tilmicosin (group 6) induced a distinct response, including significant up-regulation of *IL-1β*. Doxycycline (group 2) significantly down-regulated *IL-2* expression compared with the control group (*p* Holm ≤ 0.05). Significant down-regulation of *IL-4* was observed in group 4 (*p* Holm ≤ 0.05). *IL-6* was down-regulated in groups 2 and 4 (*p* Holm ≤ 0.05). The most consistent effect was found for *IL-8*, which was significantly down-regulated in groups 1, 2, 3, and 4 (*p* Holm ≤ 0.05). Significant down-regulation of *IL-12p40* was detected in groups 1 and 5. Interferon gene expression showed the most distinct changes: *IFN-ß* was down-regulated in groups 2, 3, 4, and 5 (*p* Holm ≤ 0.05), while *IFN-γ* was up-regulated in groups 3, 4, 5, and 6 (*p* Holm ≤ 0.05).

By day 35, splenic gene expression profiles showed partial normalization toward control levels. However, significant differences persisted for selected genes and treatment groups. Compared with the control, significant changes were detected for *IL-4* in group 6, *IL-8* in groups 3 and 6, and *IL-12p40* in groups 1 and 5 (*p* Holm ≤ 0.05). Interferon genes expression showed up-regulation of *IFN-ß* in groups 4 and 6, and down-regulation of *IFN-γ* in groups 1, 5, and 6 (*p* Holm ≤ 0.05). These results indicate that, although the strongest splenic transcriptional response occurred on day 21, selected immune-related genes remained affected by residual antimicrobial exposure at day 35.

#### 2.1.2. Caecal Mucosa

Caecal cytokine gene modulation (Figure 2) was less prominent than a splenic one in response to low-dose antibiotic administration on days 7, 21, and 35 of rearing. On day 7, no significant gene expression modulation was determined (*p* Holm ≥ 0.05). On day 21, we found significant up-regulation in the mucin gene *MUC2* in group 2 (*p* Holm ≤ 0.05). On day 35, a significant up-regulation was found in *IL-2* and *IL-8* cytokines in group 2 (*p* Holm ≤ 0.05). Overall, the gene expression modulation in caecal mucosa was much lower than in spleen, but also time-dependent. Stronger responses than in spleen were found on day 35, but not all results were statistically significant in group 2 (doxycycline).

### 2.2. Histopathological Observation of the Splenic Tissue

The histological architecture of the chicken spleen consisted of white and red pulp. The white pulp was composed of diffuse lymphoid tissue, periarterial lymphoid sheaths (PALS), and lymphatic nodules (LN). The typical statistical images of the spleen are shown in Figure 3. No statistically significant differences were observed among the experimental groups in the number of LN (Table 1). However, an increased frequency of LN atrophy was detected in groups 1 and 2 compared to the control group, suggesting potential suppression of immune function and a reduced capacity to mount an effective immune response. Although the number of lymphatic nodules remained comparable across all groups, marked qualitative alterations in splenic histopathology were observed following antibiotic administration. Lymphoid atrophy was minimal in the control group (13%, grade 1), whereas a pronounced increase was observed in antibiotic-treated birds. The highest incidence (100%) was found in groups 1 (colistin) and 2 (doxycycline), with severity reaching grade 3 in the latter. Other treatments, including flumequine (group 3) and thiamphenicol (group 4), also resulted in a high frequency of atrophy (88%), while tilmicosin (group 6) induced comparatively fewer changes (38%).

Necrotic lesions were substantially more frequent in treated groups than in controls (25%, grades 1–2). A 100% incidence of necrosis was observed in the colistin, doxycycline, and tiamulin groups, while thiamphenicol and tilmicosin groups showed necrosis in 88% of the birds. The lowest incidence among treated groups was noted for flumequine (63%), predominantly mild severity (grade 1).

Inflammatory cell infiltration was also elevated following antibiotic exposure. While detected in 38% of control animals, it increased to 63–88% across treated groups. The most pronounced infiltrates, reaching grade 3, were observed in group 4 (thiamphenicol), whereas other treatments induced mainly mild-to-moderate changes (grades 1–2). In the red pulp of the spleen, numerous erythrocytes, macrophages, and lymphocytes were observed. A tendency toward an increased occurrence of necrotic foci in the red pulp was noted in groups 1 and 2 compared to the control group; however, this finding was not statistically significant. These changes may reflect the toxic effects of antibiotic exposure and impaired splenic immune function. Additionally, occasional cytoplasmic vacuolation and vascular congestion were observed in the spleens of chickens from the experimental groups.

## 3. Discussion

In this study, residual in-feed doses of antimicrobials modulated cytokine-related gene expression in the spleen and caecal mucosa of broiler chickens. The most pronounced changes in cytokine gene expression in antimicrobial-exposed groups were observed on day 21 post-hatch in both tissues. However, the systemic transcriptional response in the spleen was more distinct than the mucosal response in the caecal mucosa.

In broilers, many immune parameters continue to develop during the first month of life. Song et al. (2021) showed that cytokine-related indices are age-dependent [25]. Several markers of peripheral and mucosal immunity increased between days 1 and 34, indicating that the immune system is still maturing during this period [25]. In the chicken spleen, Zhang et al. (2019) showed that the mRNA expression of *TLR2* and *TLR4* increased from day 1 to day 14 post-hatch and then decreased, remaining relatively stable between days 21 and 60 [26]. Similarly, *IL-2*, *IFN-γ*, and *TNF-α* mRNA levels were generally higher during the first two weeks after hatching and subsequently declined toward a more stable level [26]. These findings suggest that the early post-hatch life is characterized by stronger innate and cytokine-related immune activation in the spleen, followed by a transition toward a more stable immune state.

Day 21 post-hatch also represents an important developmental stage in chicken intestinal mucosa and microbiome maturation. In the caecum, Duangnumsawang et al. (2021) [27] reported clear age-related changes in mucosal physiology. The number of acidic goblet cells per crypt decreased from day 7 to day 21 and then remained relatively stable between days 21 and 35 [27]. The temporal development of intestinal and immune functions in broiler chickens is closely associated with increasing microbial diversity and complexity of the gut microbiota. The dynamic succession of the chicken gut microbiome has been reported to progress toward a more mature state by approximately day 21 post-hatch [28]. Preliminary unpublished data from the same project suggest that age strongly affects microbiota diversity and the relative abundance of selected bacterial taxa [29]. These findings support the interpretation that the third week post-hatch is a period of intensive intestinal and immune adaptation, during which antimicrobial perturbation may elicit particularly evident changes in the immune-related gene expression.

### 3.1. Immune-Related Gene Expression in Spleen

On day 21 post-hatch, residual in-feed antimicrobials induced the most pronounced transcriptional modulation in the spleen. However, the significant effects were gene- and treatment-specific rather than uniform across all antimicrobials. These findings are broadly consistent with previous studies in broilers showing that antimicrobials or anticoccidials administered in feed can attenuate selected systemic immune markers. For example, Lee et al. (2012) reported that medicated diets containing anticoccidials and bacitracin as the antibiotic growth promoter (AGP) decreased *IL-4*, *IL-6*, and *IL-12* expression in the spleens of 43-day-old broiler chickens [30]. In the present study, *IFN-γ* was up-regulated in the spleen on day 21 by some in-feed antimicrobials (groups 3–6), which is also consistent with the findings of Lee et al. (2012) [30]. Together, these results suggest a time-specific immunomodulatory pattern in the spleen, characterized by broad down-regulation of several cytokine-related genes accompanied by selective *IFN-γ* induction, even under residual in-feed antimicrobial exposure.

### 3.2. Immune-Related Gene Expression in Caecal Mucosa

In the caecal mucosa, the effects of residual in-feed antimicrobial exposure were much weaker than in the spleen. There is limited consensus in the literature regarding intestinal cytokine expression in chickens fed medicated diets. Lee et al. (2012) found that anticoccidial treatment, using monensin or decoquinate in combination with bacitracin as an AGP, increased the expression of *CXCLi2*, *IL-4*, *IL-6*, *IL-13*, *IL-17F*, *IFN-γ*, and *TGFβ4* in the intestinal epithelium [30]. In contrast, Pont et al. (2023) reported that bacitracin supplementation decreased several cytokine and chemokine proteins in the jejunum on day 14 and affected selected mediators in the ileum at later time points [7]. Oh et al. (2019) also showed that virginiamycin or bacitracin as antibiotic growth promoters dampened pathogen-induced intestinal cytokine responses in chickens challenged with *Eimeria maxima* and *Clostridium perfringens* [8]. Compared with these studies, our results indicate that residual antimicrobial concentrations had a more limited effect on caecal cytokine gene expression.

### 3.3. Intestinal Barrier-Related Gene Expression in Caecal Mucosa

In the caecal mucosa, the most evident barrier-related response was selective up-regulation of *MUC2* on day 21 in group 2. In poultry, the intestinal mucus layer is a key component of the mucosal innate defense. It is produced by goblet cells, forms the first physical barrier against luminal microbes, and contributes to the host–microbiota homeostasis [27]. *MUC2* encodes secretory gel-forming mucin. Schokker et al. (2017) investigated an early-life microbiota perturbation induced by amoxicillin treatment on day 1 post-hatch and reported increased *MUC2* expression on day 5 [31]. In this context, the significant *MUC2* up-regulation observed in the present study may reflect an adaptive response of the caecal mucosal barrier to antimicrobial-induced disturbance of the intestinal environment.

### 3.4. Spleen Histopathology

The spleen is the largest secondary lymphoid organ in chickens and plays a central role in systemic immune surveillance and immune responses. It is composed of white and red pulp, which differ in structure and function. The white pulp contains organized lymphoid compartments, including T lymphocytes in the periarteriolar lymphoid sheath (PALS), B lymphocytes in the periellipsoid lymphoid sheath (PELS), and germinal centers involved in B-cell proliferation and differentiation. The red pulp, composed of splenic sinuses and cords, is mainly responsible for blood filtration and the removal of circulating pathogens and cellular debris [32]. In the present study, histopathological evaluation of the broiler spleens after residual in-feed antimicrobial exposure revealed consistent morphological alterations, including lymphoid atrophy, necrosis, inflammatory cell infiltration, and occasional cytoplasmic vacuolation and vascular congestion.

Although the number of lymphoid nodules did not differ significantly among the experimental groups, marked differences in their size and structural integrity were evident. Most nodules were small, and germinal centers frequently contained only single macrophages and lymphoblasts, suggesting reduced lymphoid activity and impaired follicular responses. The most pronounced atrophy of lymphoid nodules was observed in groups 1 and 2, which differed significantly from the control group. Similar lymphoid depletion has been reported under various stress conditions, such as heat stress in Japanese quail [33], indicating that different environmental and pharmacological stressors may converge on common immunosuppressive pathways. A reduction in lymphocyte density within splenic follicles may reflect impairment of cellular components involved in innate and adaptive immunity, as previously suggested [34].

Furthermore, the increased occurrence of the necrotic cells observed in several experimental groups, particularly groups 1, 2, and 5, indicates tissue damage that may compromise splenic immune function. Necrotic lesions within splenic lymphoid structures may compromise splenic immune function by disturbing lymphoid architecture and reducing the cellular integrity required for coordinated immune responses. Similar spleen lesions, including increased necrotic cells, splenocyte apoptosis, cell-cycle arrest, and changes in T-cell subsets, were reported in broiler chickens exposed to aflatoxin-contaminated feed and were associated with immunosuppression [35]. The presence of inflammatory infiltrates and vascular alterations further supports the presence of tissue remodeling and immune dysregulation.

Importantly, these histopathological findings may be linked to the immunomodulatory effects of residual in-feed antimicrobial exposure. Low-dose antimicrobials can alter the composition and diversity of the gut microbiota, which plays an important role in shaping systemic immune responses. Disruption of the microbiota–gut–immune axis may therefore contribute to altered cytokine signaling, including pathways involving IFN-γ, a key mediator of cellular immunity. In the present study, changes in IFN-γ expression may be associated with an altered activation of macrophages and T lymphocytes, potentially contributing to the lymphoid depletion and structural changes observed in the spleen. Collectively, our results indicate that the residual in-feed antimicrobial exposure can induce measurable structural alterations in the spleen, reflecting disturbed lymphoid homeostasis and altered immune competence. These findings highlight the need for careful evaluation of residual antimicrobial exposure in poultry production, particularly in the context of immune system development and disease resistance.

### 3.5. Effects of Antimicrobials

Overall, our findings may be interpreted in light of Niewold’s concept of the mode of action of AGPs [36]. This concept proposes that low doses of antimicrobials may influence host physiology not only through antibacterial activity, but also by modulating inflammatory responses. According to this hypothesis, low-dose antimicrobials may interact with the intestinal mucosa and reduce the production of catabolic inflammatory mediators by intestinal immune cells [36]. In the present study, the down-regulation of several splenic cytokine-related genes, together with transient changes in caecal cytokine and mucin gene expression, suggests that chronic host responses to residual antimicrobial exposure should be considered in addition to antimicrobial effects on luminal bacteria.

## 4. Materials and Methods

### 4.1. Study Design

The study design included six treatment groups and one control group. The treatment groups were fed diets containing residual doses of antibiotics (colistin, doxycycline, flumequine, thiamphenicol, tiamulin, and tilmicosin). The doses of VMPs were calculated as 2% of the maximum approved concentration according to summaries of product characteristics (SmPCs). When available, premixes authorized in Poland were used for this purpose with only exception of flumequine (Table 2). The antibiotic-contaminated feed was delivered throughout the whole rearing period. Table 3 presents basic feed composition, and Table 4 presents content of nutrients in the feed. The experiment was performed as follows: one-day-old female broiler chicks (line Ross 308) were randomly distributed to 42 pens (12 animals/pen, 504 animals in total). Each group included six pens. On days 7, 21, and 35 of rearing, 8 animals per group were humanely euthanized and dissected. The samples of spleen and mucosal scrapings were collected and preserved in RNA stabilization solution (fixRNA, Eurx, Gdansk, Poland). For histopathological examination representative pieces of spleen samples were collected and fixed in 10% neutral buffered formalin for at least 48 h. The animal procedures were approved by the Local Ethics Committee for Animal Experiments in Kraków, Poland (approval no. 650/2022 dated 14 July 2022).

### 4.2. RNA Isolation and RT-qPCR

Total RNA was isolated using Universal RNA Purification Kit (EURx, Gdansk, Poland). The concentration and purity of the eluted RNA was measured with the NanoDrop 2000 (Thermo Scientific/NanoDrop Technologies, Wilmington, NC, USA). The total RNA was assessed for integrity by agarose gel electrophoresis. Gene expression analysis was performed using 2-step RT-qPCR. The amount of 3.5 µg of total RNA was reversely transcribed with smART First Strand cDNA Synthesis Kit (EURx, Gdansk, Poland). Obtained cDNA was diluted to a working concentration of 70 ng/µL and stored at −20 °C. RT-qPCR reactions were run using SG onTaq qPCR Master Mix (EURx, Gdansk, Poland). RT-qPCR reaction mix included: 6.25 µL of SG onTaq qPCR Master Mix, 1 µM of each oligonucleotide primer and 2 µL of diluted cDNA. The reaction volume was adjusted to 12.5 µL with nuclease-free water. Each RT-qPCR reaction was performed in two technical replicates. Thermal cycling began with an initial denaturation step at 95 °C for 5 min, followed by 40 amplification cycles comprising denaturation at 95 °C for 15 s, annealing at 58 °C for 15 s, and extension at 72 °C for 45 s. Fluorescence signals were recorded after each extension phase. A melting curve analysis was performed by increasing the temperature to 98 °C while continuously monitoring the fluorescence of the dissociating amplicons. Sequences of oligonucleotide primers used to amplify the immune-related genes are listed in Table 5.

The relative gene expression analysis was performed on both tissues with the panel of interleukine (IL) genes, including: *IL-1ß*, *IL-2*, *IL-4*, *IL-6*, *IL-8*, and *IL-12p40*. Additionally, in caecal mucosa two mucin (MUC) genes were analyzed: *MUC2* and *MUC6*. In spleen, the expression of two interferons (IFN) was determined: *IFN-ß* and *IFN-γ*. The reference genes were: *ACTB* and *G6PDH*. The relative gene expression was performed with ddCt method, and the results were presented as log2 Fold Change (Log2FC).

Statistical analyses were performed separately for each tissue, sampling day, and gene. For statistical evaluation only averaged biological replicates were used. Because the data did not consistently meet the assumptions of normality, differences among groups were assessed using the Kruskal–Wallis test. Post hoc comparisons between each antimicrobial-treated group and the control group were performed using Dunn’s test. To account for multiple post hoc comparisons, *p*-values were adjusted using the Holm method. Differences were considered statistically significant at Holm-adjusted *p* ≤ 0.05. JASP software was used for statistical evaluation (JASP Team, 2025, version 0.95.3).

### 4.3. Histopathology

The spleen samples were fixed in 10% neutral buffered formalin for at least 48 h. Subsequently, they were routinely processed using an automated vacuum tissue processor (ASP6025 S, Leica, Nussloch, Germany). Tissues were embedded in paraffin (HistoCore Arcadia H, Leica, Nussloch, Germany), and paraffin blocks were cut to 3 μm thickness sections using a microtome (HistoCore MULTICUT, Leica, Nussloch, Germany). Sections were stained with hematoxylin and eosin (ST Infinity Kit, Leica, Nussloch, Germany). The number of lymphatic follicles and the cellular composition of the red pulp were counted on the cross-section of the spleen. Microscopic analysis was conducted using a Nikon Ci-L light microscope with a Nikon DS-Fi3 5.9 MPix digital camera (version 5.42.00; Nikon Corporation, Tokyo, Japan). Image analysis was performed with the NIS-Elements BR software (Nikon Corporation, Tokyo, Japan). To assess the severity of histopathological changes, ten randomly selected microscopic fields per sample were assessed, and lesions were graded as absent (0), minimal (1), moderate (2), or severe (3). Lymphatic nodules were counted in five randomly selected microscopic fields at 40× magnification, corresponding to a total analyzed area of 25 mm^2^. The results were expressed as the number of nodules per 25 mm^2^. Histopathological sections were independently evaluated in a blinded manner by two experienced observers using a standardized scoring system. Lesions were graded according to established parameters to reduce inter-observer variability and ensure reproducibility.

## 5. Conclusions

Residual in-feed exposure to antimicrobial-modulated immune-related gene expression in broiler chickens, despite being far below therapeutic levels. The strongest effects were observed on day 21 post-hatch, indicating that the third week of life may represent a particularly sensitive period for antimicrobial-driven immune modulation. The response was tissue-specific: the spleen showed a broader and predominantly down-regulated cytokine profile, whereas the caecal mucosa showed a weaker but mainly up-regulated cytokine response, accompanied by increased expression of the mucin genes. These findings indicate that feed cross-contamination with antimicrobials may affect both systemic and mucosal immune regulation, as well as intestinal barrier-related responses. The down-regulation of the cytokine expression in the spleen may be associated with lymphoid atrophy and reduced germinal center activity, suggesting a link between antimicrobial-induced transcriptional changes and impaired splenic immune architecture. Overall, this study shows that residual antimicrobial exposure should be considered a biologically relevant factor in poultry production and feed safety assessment.

## Figures and Tables

**Figure 1:**
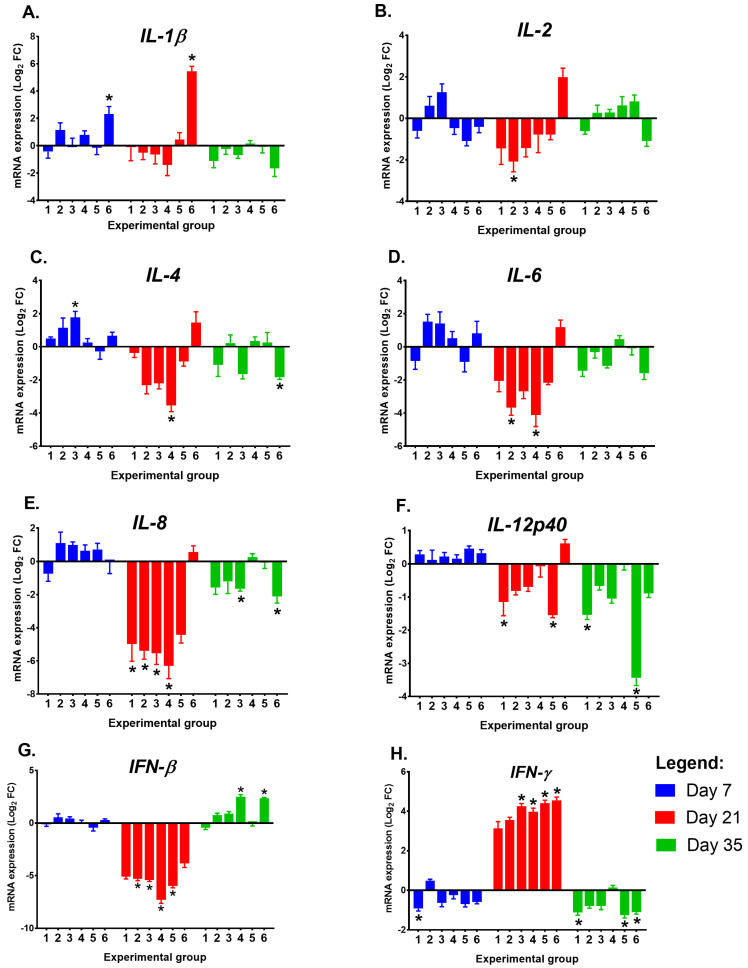
Immune-related gene expression in the spleen of chickens fed diets containing residual concentrations of antibiotics (1—colistin, 2—doxycycline, 3—flumequine, 4—thiamphenicol, 5—tiamulin, or 6—tilmicosin), compared with the antibiotic-free control group on days 7 (blue), 21 (red), and 35 (green) of rearing. The gene expression was determined by RT–qPCR and normalized to the reference genes *ACTB* and *G6PDH*. The relative gene expression was performed with ddCt method, and the results were presented as log2 Fold Change (Log2FC). Statistically significant differences in experimental groups vs. the control are indicated by an asterisk (Kruskal–Wallis test with Dunn’s post hoc test, Holm-adjusted *p* ≤ 0.05).

**Figure 2:**
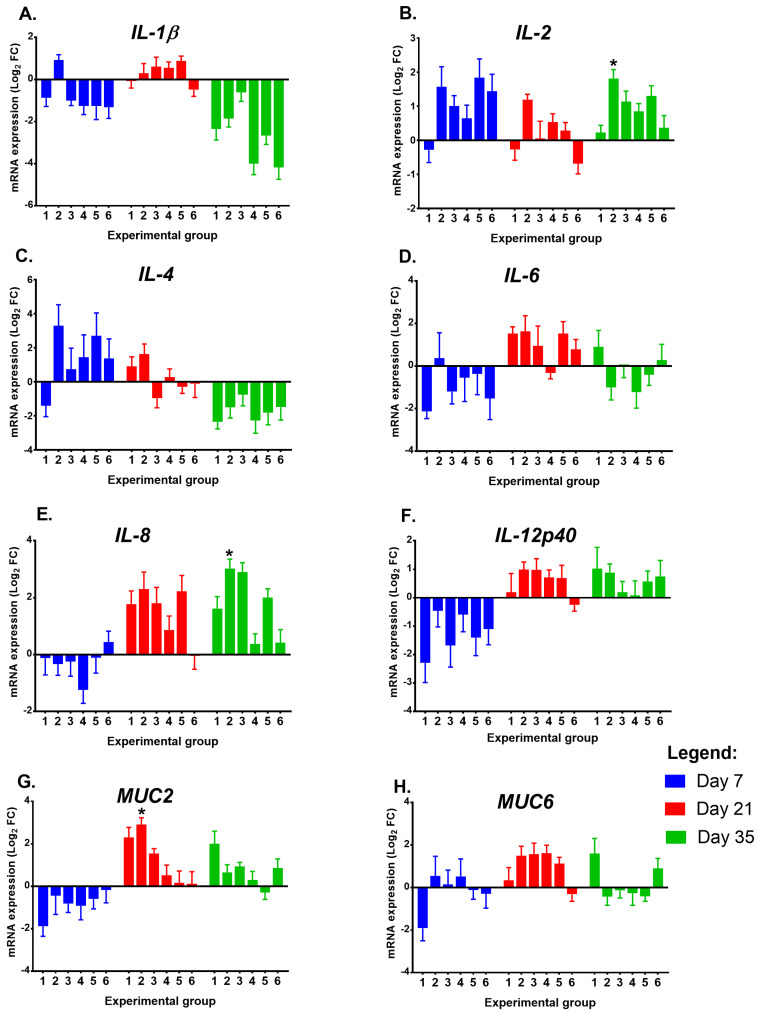
Immune-related gene expression in the caecum mucosa of chickens fed diets containing residual concentrations of antibiotics (1—colistin, 2—doxycycline, 3—flumequine, 4—thiamphenicol, 5—tiamulin, or 6—tilmicosin), compared with the antibiotic-free control group on days 7 (blue), 21 (red), and 35 (green) of rearing. The gene expression was determined by RT–qPCR and normalized to the reference genes *ACTB* and *G6PDH*. The relative gene expression was performed with ddCt method, and the results were presented as log2 Fold Change (Log2FC). Statistically significant differences in experimental groups vs. the control are indicated by an asterisk (Kruskal–Wallis test with Dunn’s post hoc test, Holm-adjusted *p* ≤ 0.05).

**Figure 3:**
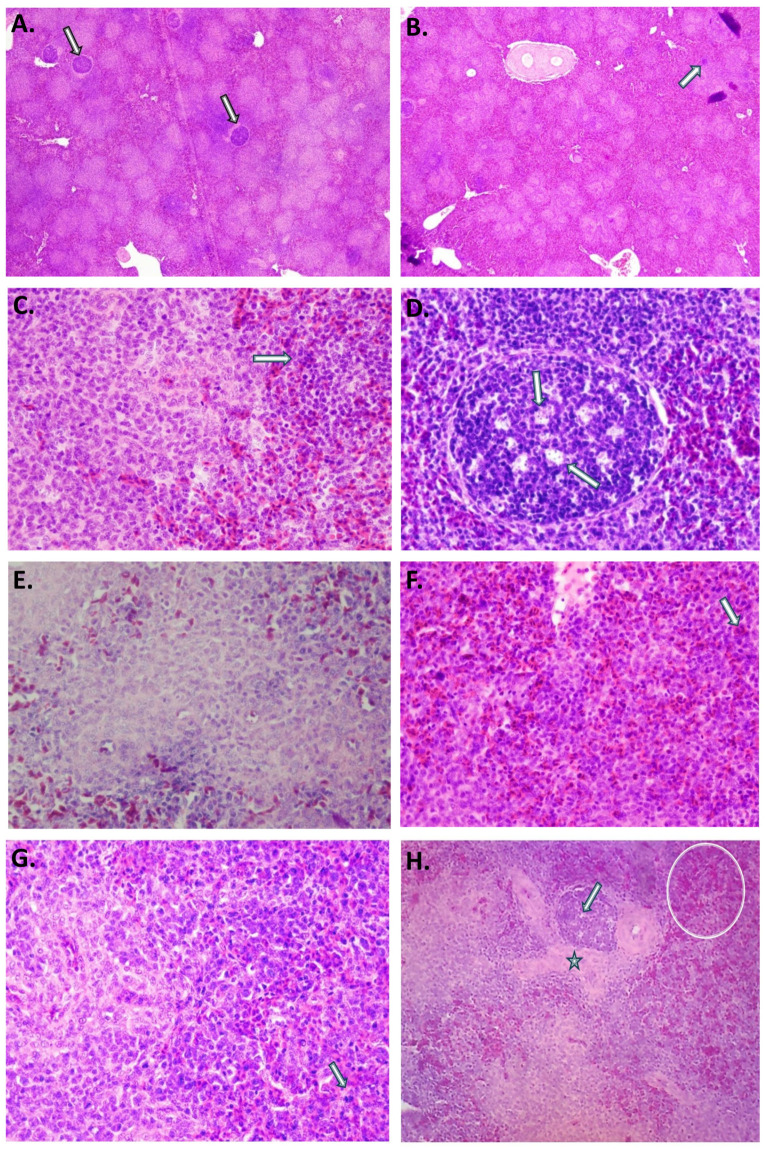
Histological images of the chicken spleen on day 35 of age (HE staining). (**A**) The control group (0—antibiotic-free) showing normal structure of periarterial lymphoid tissue and splenic nodule (arrows), mag. 40×; (**B**) group 2 (doxycycline) showing atrophy of lymph nodules (arrows), mag. 40×; (**C**) group 3 (flumequine) showing migration of lymphocytes from PALS to the red pulp (arrow), mag. 400×; (**D**) group 3 (flumequine) showing apoptotic bodies in the germinal center of lymph nodes (arrows), mag. 400×; (**E**) group 2 (doxycycline) showing focus of lymphoid cell necrosis, mag. 400×; (**F**) group 2 (doxycycline) showing heterophil (arrow), mag. 400×; (**G**) group 4 (thiamphenicol) showing macrophage (arrow), mag. 400×; (**H**) group 5 (tiamulin) showing hyperemia (circle), fibrosis (asterisk), apoptotic bodies in the lymph node (arrow), mag. 100×.

**Table 1:** Histopathological changes in chicken spleen following experimental in-feed administration of low-dose antibiotics.

Group/Antibiotic	Number of LN	Histopathological Changes					
		Atrophy of LN		Necrosis		Inflammatory Infiltrates	
		n (%)	Grade *	n (%)	Grade *	n (%)	Grade *
(0) Control	13 ± 5.0	13	1	25	1 to 2	38	1 to 2
(1) Colistin	19 ± 4.7	100	1 to 2	100	1 to 2	63	1 to 2
(2) Doxycycline	14 ± 4.7	100	1 to 3	100	1 to 3	75	1 to 2
(3) Flumequine	14 ± 4.2	88	1 to 2	63	1	88	1 to 2
(4) Thiamphenicol	17 ± 5.3	88	1 to 2	88	1 to 2	88	1 to 3
(5) Tiamulin	15 ± 4.7	50	1 to 3	100	1 to 2	88	1 to 2
(6) Tilmicosin	13 ± 5.2	38	1 to 2	88	1	88	1 to 2

LN—lymphatic nodules, * Histopathology grade: absent 0, minimal 1, moderate 2, severe 3.

**Table 2:** The dosage of the antibiotic residues for the experimental groups.

Group No	Group Name	The Name of VMP That Was the Basis for Dose Calculation	Maximum Concentration in Feed According to VMP’s SmPC [mg/kg]	Antimicrobial Concentration in Experimental Feed [mg/kg]
0	Control	N/A	N/A	-
1	Colistin	Apsamix Colistina	300	6
2	Doxycycline	Pulmodox	250	5
3	Flumequine	Flumesyva	2400	15
4	Thiamphenicol	Tirsan	1000	20
5	Tiamulin	Vetmulin	500	10
6	Tilmicosin	Tilmovet	400	8

**Table 3:** Composition of the basic feed mixtures.

Ingredient [g/kg]	Starter, 1–21 d of Age	Grower–Finisher, 22–35 d of Age
Maize	585.1	614.45
Soybean meal	360	310
Soybean oil	20	38
Limestone	11	13
Sodium chloride	3.3	3.7
Monocalcium phosphate	14	13.5
DL-Methionine	2.1	2.1
L-Lysine hydrochloride	0.5	1.9
L-Treonine	-	0.35
Vitamin–mineral premix *	4	3

* Each kilogram of the vitamin–mineral premix contained the following: vitamin A—3,666,700 IU; vitamin D3—833,340 IU; 25-dihydroxycholecalciferol—6.94 mg; vitamin E—16,677 mg; vitamin K3—833 mg; vitamin B1—667 mg; vitamin B2—2333 mg; vitamin B6—1333 mg; vitamin B12—7 mg; Ca-pantothenate—4537.4 mg; niacin—13,333 mg; folic acid—333 mg; biotin—67 mg; choline chloride—100,000 mg; manganese—23,333 mg; zinc—31,666 mg; iron—15,000 mg; copper—6667 mg; iodine—200 mg; selenium—117 mg.

**Table 4:** Metabolizable energy and content of nutrients in basic feed mixtures.

Analyzed Chemical Composition [g/kg]	Starter, Days 1–21	Grower–Finisher, Days 22–35
Metabolizable energy (MJ/kg), calculated	12.15	12.62
Dry matter	879.9	892.4
Crude protein	209.3	197.4
Crude fat	56.6	74.2
Crude ash	51.5	50.0
Crude fiber	23.8	20.0
Calcium	8.09	8.47
Aspartic acid	223.7	198.3
Threonine	82.6	77.2
Serine	118.9	107.4
Glutamic acid	410.1	370
Proline	124.5	115.4
Glycine	98.1	87.7
Alanine	113.8	105.2
Valine	101.4	92.8
Isoleucine	94.4	85.1
Leucine	187.3	173.8
Tyrosine	82.1	74.4
Phenylalanine	111.3	101.3
Histidine	62.1	55.8
Lysine	128.9	126.7
Arginine	141.8	121.7
Cysteine	33.6	31.4
Methionine	55.4	52.2
Tryptophan	25.5	15.5

**Table 5:** Oligonucleotide primers for RT-qPCR reactions.

Gene Symbol	Gene Name	Primer Sequence (5′→3′)	Ref.
*ACTB*	Actin, beta	F: CACAGATCATGTTTGAGACCTT R: CATCACAATACCAGTGGTACG	[37]
*G6PDH*	Glucose-6-Phosphate Dehydrogenase	F: CGGGAACCAAATGCACTTCGT R: GGCTGCCGTAGAGGTATGGGA	[37]
*IL1-β*	Interleukin 1 beta	F: GGAGGTTTTTGAGCCCGTC R: TCGAAGATGTCGAAGGACTG	[38]
*IL-2*	Interleukin 2	F: GCTTATGGAGCATCTCTATCATCA R: GGTGCACTCCTGGGTCTC	[39]
*IL-4*	Interleukin 4	F: GCTCTCAGTGCCGCTGATG R: GGAAACCTCTCCCTGGATGTC	[40]
*IL-6*	Interleukin 6	F: AGGACGAGATGTGCAAGAAGTTC R: TTGGGCAGGTTGAGGTTGTT	[41]
*IL-8*	Interleukin 8	F: AAGGATGGAAGAGAGGTGTGCTT R: GCTGAGCCTTGGCCATAAGT	[40]
*IL-12p40*	Interleukin 12p40	F: TTGCCGAAGAGCACCAGCCG R: CGGTGTGCTCCAGGTCTTGGG	[42]
*IFN-β*	Interferon beta	F: ACCAGATCCAGCATTACATCCA R: CGCGTGCCTTGGTTTACG	[40]
*IFN*-γ	Interferon gamma	F: ACACTGACAAGTCAAAGCCGC R: AGTCGTTCATCGGGAGCTTG	[42]
*MUC2*	Mucin 2	F: ATGCGATGTTAACACAGGACTC R: GTGGAGCACAGCAGACTTTG	[43]
*MUC6*	Mucin 6	F: TTCAACATTCAGTTCCGCCG R: TTGATGACACCGACACTCCT	[44]

## Data Availability

The data supporting the findings of this study are available from the corresponding author upon reasonable request.

## Abbreviations

The following abbreviations are used in this manuscript:

AGP	Antibiotic Growth Promoter
ACTB	Beta-Actin
CD3	Cluster of Differentiation 3
CD4	Cluster of Differentiation 4
CD8	Cluster of Differentiation 8
cDNA	Complementary DNA
CXCLi2	C-X-C Motif Chemokine Ligand i2
ddCt	Delta-Delta Cycle Threshold Method
ELISA	Enzyme-Linked Immunosorbent Assay
EU	European Union
G6PDH	Glucose-6-Phosphate Dehydrogenase
HE	Haematoxylin and Eosin
IFN-β	Interferon Beta
IFN-γ	Interferon Gamma
IL-1β	Interleukin 1 Beta
IL-2	Interleukin 2
IL-4	Interleukin 4
IL-6	Interleukin 6
IL-8	Interleukin 8
IL-12p40	Interleukin 12 p40 Subunit
LN	Lymphoid Nodule
Log2FC	Log2 Fold Change
LPS	Lipopolysaccharide
MAPK	Mitogen-Activated Protein Kinase
M-CSF	Macrophage Colony-Stimulating Factor
MIP-1β	Macrophage Inflammatory Protein 1 Beta
MIP-3α	Macrophage Inflammatory Protein 3 Alpha
MUC2	Mucin 2
MUC6	Mucin 6
NF-κB	Nuclear Factor Kappa B
PALS	Periarteriolar Lymphoid Sheath
PELS	Periellipsoid Lymphoid Sheath
RT-qPCR	Reverse Transcription Quantitative Polymerase Chain Reaction
SmPCs	Summary of Product Characteristics
TGFβ4	Transforming Growth Factor Beta 4
TLR2	Toll-Like Receptor 2
TLR4	Toll-Like Receptor 4
TNF-α	Tumor Necrosis Factor Alpha
VEGF	Vascular Endothelial Growth Factor
VMP	Veterinary Medicinal Product
